# Primates, Provisioning and Plants: Impacts of Human Cultural Behaviours on Primate Ecological Functions

**DOI:** 10.1371/journal.pone.0140961

**Published:** 2015-11-04

**Authors:** Asmita Sengupta, Kim R. McConkey, Sindhu Radhakrishna

**Affiliations:** School of Natural Sciences and Engineering, National Institute of Advanced Studies, Indian Institute of Science Campus, Bangalore, India; Sichuan University, CHINA

## Abstract

Human provisioning of wildlife with food is a widespread global practice that occurs in multiple socio-cultural circumstances. Provisioning may indirectly alter ecosystem functioning through changes in the eco-ethology of animals, but few studies have quantified this aspect. Provisioning of primates by humans is known to impact their activity budgets, diets and ranging patterns. Primates are also keystone species in tropical forests through their role as seed dispersers; yet there is no information on how provisioning might affect primate ecological functions. The rhesus macaque is a major human-commensal species but is also an important seed disperser in the wild. In this study, we investigated the potential impacts of provisioning on the role of rhesus macaques as seed dispersers in the Buxa Tiger Reserve, India. We studied a troop of macaques which were provisioned for a part of the year and were dependent on natural resources for the rest. We observed feeding behaviour, seed handling techniques and ranging patterns of the macaques and monitored availability of wild fruits. Irrespective of fruit availability, frugivory and seed dispersal activities decreased when the macaques were provisioned. Provisioned macaques also had shortened daily ranges implying shorter dispersal distances. Finally, during provisioning periods, seeds were deposited on tarmac roads that were unconducive for germination. Provisioning promotes human-primate conflict, as commensal primates are often involved in aggressive encounters with humans over resources, leading to negative consequences for both parties involved. Preventing or curbing provisioning is not an easy task as feeding wild animals is a socio-cultural tradition across much of South and South-East Asia, including India. We recommend the initiation of literacy programmes that educate lay citizens about the ill-effects of provisioning and strongly caution them against the practice.

## Introduction

Human provisioning of wildlife with food is a widespread global practice that occurs in multiple socio-cultural contexts. Food is often provided voluntarily to animals within human residential neighbourhoods and temples as a religious ritual or a cultural convention or in wildlife tourism spots through feed stations [1–3]. Provisioning may also be inadvertent when farms, plantations, home gardens, waste food dumps and kitchens become sources of food for animals [4]. Provisioned food resources tend to be high-calorie, easily digestible, spatio-temporally predictable and available in greater proportions than natural resources in a given area [5, 6]. The effects of such human food subsidies on wildlife species are varied and may range from increase in body size and fertility at the individual level to altered foraging and migratory behaviour at the population level [3]. Provisioning is hence predicted to alter or modify ecosystem services and evolutionary processes through its impact on food webs and community assemblages [2, 3].

Throughout history, humans and primates have co-existed in diverse cultures and contexts and provisioning wild primates is a socio-religious tradition in many Asian countries, including India [1, 7]. It has been suggested that primate supplemental feeding results in faster growth of individuals, attainment of early sexual maturity, longer survival and reproduction at shorter intervals [8]. Provisioned primate troops have been observed to decrease their consumption of natural plant parts, their mean daily and home ranges and spend more time resting and less time feeding and foraging [6, 9–13]. Provisioning may also heighten intra- and inter group aggression, alter within-group social dynamics, increase infant mortality risks and promote group fission [14–18]. Although the importance of food resources as drivers for animal ecology and behaviour is indisputable and it has been recognized that provisioning may indirectly alter ecosystem functioning through changes in behaviour and abundance of animals [19], few studies have actually quantified this aspect [20–22].

Our study addresses this lacuna in research through an examination of the effects of provisioning on wild primates and the consequences of this on the ecosystem process of seed dispersal. Seed dispersal is an important ecological process which removes seeds from parent trees, thereby enabling them to escape competition over the same resources, and deposits them at sites favourable for germination, thereby increasing gene flow [23]. It is the principal driver of tropical forest recruitment and the recolonization and restoration of degraded habitats [24, 25]. Frugivorous primates are important seed dispersers for a broad range of species and changes in primate feeding ecology critically impacts seed deposition and germination [26–29].

The rhesus macaque is a dietarily flexible primate species that inhabits a variety of habitats ranging from tropical moist and dry deciduous forests to temperate coniferous and mixed forests, scrub jungles and human settlements across south and south east Asia [30, 31]. In our previous study on a troop of non-provisioned rhesus macaques (Troop C) at the Buxa Tiger Reserve (BTR) in northern India, we observed that the macaque is an important seed disperser for 41 plant species in the area. Almost 96% of the handled seeds were undamaged and macaque seed handling either had positive or neutral effect on seed germination. About 50% of monitored seeds deposited *in situ* germinated while 22% established seedlings by the end of a year, thereby indicating that rhesus macaques can be effective seed dispersers [32]. Within the same Reserve, some troops that resided near the highway were routinely provisioned by tourists during some parts of the year, while for the remaining period, they were dependent on natural resources. As this provided an ideal, natural situation to examine how provisioning may affect the ecological functions of primates, we conducted a study to investigate the impacts of provisioning on the role of rhesus macaques as seed dispersers. We addressed the following specific questions: (a) How does provisioning impact macaque frugivory and ranging patterns? (b) How does provisioning affect dispersal activity, i.e. number of dispersal events and sites of seed deposition? We predicted that:

1. Rhesus macaques would decrease consumption of forest food resources during provisioning periods
2. Macaque day ranges would be smaller during provisioning periods.
3. Macaque seed dispersal events would decrease during provisioning months.
4. During provisioning periods, the majority of seed deposition sites would be unconducive for germination.

## Methods

### Study Area

**Fig 1:**
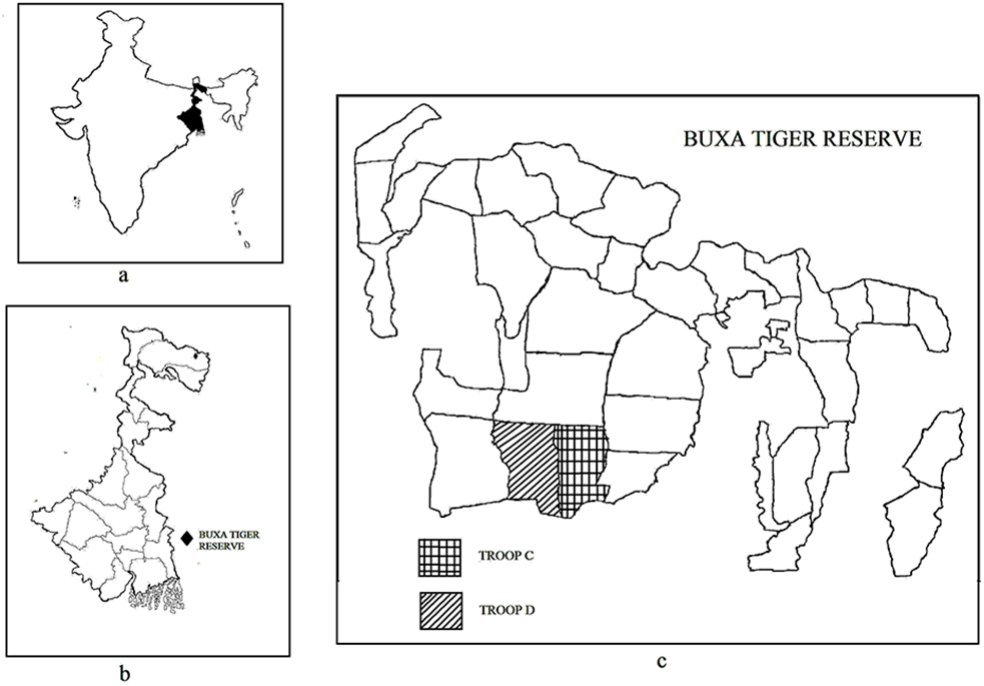
Location of the study site. a. Location of West Bengal within India, b. Location of BTR within West Bengal, c. Location of Damanpur Block within BTR.

We obtained necessary research permits from the West Bengal Forest Department, India. Data collection was carried out at the Buxa Tiger Reserve (BTR henceforth; 26°30’-23°50’N, 89°25’-89°55’E), a protected forest area in the State of West Bengal, India (Fig 1) from October, 2013 to September, 2014. BTR encompasses an area of 761 sq.km and the elevation ranges from 60 to 1750 m [33]. Temperatures vary between 12 and 32°C and the mean annual rainfall is 4100 mm [33]. The various vegetation types in BTR include tropical moist deciduous, evergreen, semi-evergreen, scrub and riverine forests apart from grasslands and plantations [34].

### Study Troop

We observed a troop of 64 rhesus macaque individuals (15 adult males, 22 adult females, 11 juvenile males, 13 juvenile females and 3 infants), referred to as Troop D, within the Damanpur Block located within the buffer zone of BTR. The home range of the study troop (mean: 55.4 ha; range: 20–72 ha, N = 12 months) included patches of natural forests, mixed species plantations and residential settlements. State Highway 12A and National Highway 31C passed through this area. During the main tourism months- October, December, January and April, there was heavy traffic on the Highways. The study troop was primarily provisioned in the vicinity of a teashop that was situated near the juncture of State and National Highways 12A and 31C. Macaques were fed by tourists who threw food (cookies, boiled chickpeas, bananas, chips, cake) from passing vehicles and by visitors to the teashop who provided hand-outs of bread and cookies. Additionally, the macaques also fed from the waste food dumps located near the teashop and on kitchen wastes generated from adjacent residential blocks. We defined all such food resources procured from anthropogenic sources as ‘provisioned food’. BTR was closed to visitors between 15th June and 15th September.

### Dietary observations and seed handling mechanisms

We followed the macaques of Troop D from their waking site to their sleeping tree for 10 days each month (12 hrs a day; 5 days each in the 2nd and 3rd weeks of each month) and noted down their feeding activities using a 30‐min interval scan sampling method [35]. The duration of each scan was 15 mins and we waited for 15 minutes before beginning the next scan. We identified all the individuals of the troop. To ensure that we did not make multiple observations on the same individual within the same scan, we always scanned the troop from left to right. We noted the following activities of all the individuals which were visible within the duration of the scan: moving, resting, social interactions and feeding. We recorded the activities of individuals immediately after we detected them (within 2 seconds). When we observed the macaques to be feeding, we noted the food item (plant species and part thereof: fruit, leaf, flower, shoot; insects; fungi; provisioned food) being consumed. For each month, we calculated degree of frugivory as the proportion of feeding scans in which fruits were eaten expressed as monthly percentages and the degree of provisioning as proportion of feeding scans in which the macaques were provisioned expressed as monthly percentages.

If in any scan, we recorded the macaques to be feeding on fruits, after the completion of the scan, we employed focal animal [35] sampling on a randomly chosen adult individual lasting 30 minutes. During this period, we conducted continuous recording [36] of fruit-feeding behaviours to make detailed observations on the part of the fruit consumed (whole fruit, pulp, seed) and the way seeds were handled. The state of ripeness of the fruits (ripe/unripe) were noted and the following seed handling mechanisms were identified: swallowed (when the entire fruit was ingested, digested and the seeds egested intact), spat out (when the fruit was taken into the mouth, mostly stored in cheek pouches, cleaned of the pulp and the seeds expectorated) and destroyed (when seeds were consistently crunched by macaques or if the fruits of those species were consumed in an unripe state) [32, 37, 38]. We began the next scan after the focal animal sampling was over. Focal animals were sampled without replacement [39] to ensure that we did not sample the same individual repeatedly for subsequent observations on seed handling mechanisms. Between scans, we visually examined the remnants of fruits/seeds beneath the feeding trees to confirm if seeds were spat out clean or if they were crunched. We also opportunistically collected fresh fecal material from the troop individuals and examined them by teasing the sample apart with the help of a pair of small twigs after placing the sample on a leaf. We then counted the number of seeds, identified them and checked if they were intact or crunched. The daily ranges of the macaques were logged with the help of a hand-held GPS unit (Garmin Etrex 30). We recorded the co-ordinates of the location of the troop at an interval of 15 minutes throughout the period of observation. We then used GPS TrackMaker version 13.9 to measure the total route distance travelled in a day and calculated the troop’s mean daily range for each month (N = 10 days).

### Dispersal Events and Seed Deposition Sites

Rhesus macaques may disperse seeds via fecal matter or through expectoration [32]. Hence, we calculated the number of Dispersal Events (DE) for both kinds of seed handling mechanisms. For fecal seeds, DE was defined as the number of fecal samples containing seeds, for each month and for each plant species [37, 40]. We defined DE for spat out seeds as the number of fruit-feeding scans in which we observed the macaques to spit out the seeds of plant species. As equal numbers of fecal samples were not collected across all the months, we also calculated DE ratios (DE_r_) for fecal seeds as the ratio of number of fecal samples in which seeds of at least one species was found to the total number of fecal samples collected for each month [37, 40]. For spat out seeds, the DE_r_ was calculated as ratio of number of scans in which macaques were observed to spit out the seeds of at least one species to the total number of fruit feeding scans. We noted the habitat type (roads, primary forests, plantations) where seed deposition occurred and calculated the ratio of seed deposition sites observed on the road to the total number of deposition sites (DS_r_).

### Fruit Availability Index

We assessed fruit availability in the home range of the study troop using four 500 m-long transects. Two of these were oriented in the North-South direction while the remaining two were oriented East-West. Each transect was 20 m in width, and the four transects covered 15.7% of the home range area. We marked all trees with DBH (diameter at breast height) ≥ 10 cm and lianas along these transects and recorded 788 trees belonging to 64 species. Basal area of each tree (B) was calculated using the following formula:

$$B\;=\;{\left(0.5\;*\;dbh\right)}^{2}\;*\;\pi$$

Every month, we monitored the fruit availability of the trees and lianas. Based on the percentage of crown area covered by fruit, we ranked trees and lianas on a 5-point scale where a score of score of 0 implied no fruit and 1 2, 3 and 4 implied 1–25%, 26–50%, 51–75% and ≥ 76% of the crown area covered by fruit respectively [38]. A Fruit Availability Index (FAI) for each month was calculated using the following formula:

$$FAI=\sum_{i=1}^{n}D_{i}\;B_{i}P_{im}$$

Where *D*_*i*_ is the density of a particular species *i* in the home range, *B*_*i*_ is the mean basal area of trees of species *i* (cm^2^), *P*_*im*_ is the mean phenology score of fruit in species *i* in a given month *m* and *n* is the number of species considered in the study [38].

### Statistical analyses

We used linear modelling to investigate the relationship between (i) degree of frugivory, degree of provisioning and FAI and (ii) degree of provisioning and DE_r_ and DS_r_. We used Chi-square test [39] to determine if the percentage of species which had their seeds spat out differed significantly from those which were swallowed or destroyed. We used binomial tests [39] to determine if (i) DE_r_ was significantly lower or higher than mean DE_r_ in some months and (ii) the months DS_r_ was significantly higher or lower than mean DS_r_. All statistical analyses were carried out using R version 3.1.1 [41].

## Results

We obtained 2880 scans (720 hrs) and 480 focal animal samples (240 hrs) on macaque feeding behaviour over 12 months. We also collected 367 fresh macaque fecal samples.

In the month of May, the number of tourists was minimal; therefore macaques had no access to tourist provisioning during this month apart from the period when BTR was closed to visitors (June to September). Hence we considered May to September as the ‘non-provisioning period’ and October to April as the ‘provisioning period’

### Fruit availability, degree of provisioning and degree of frugivory

**Fig 2:**
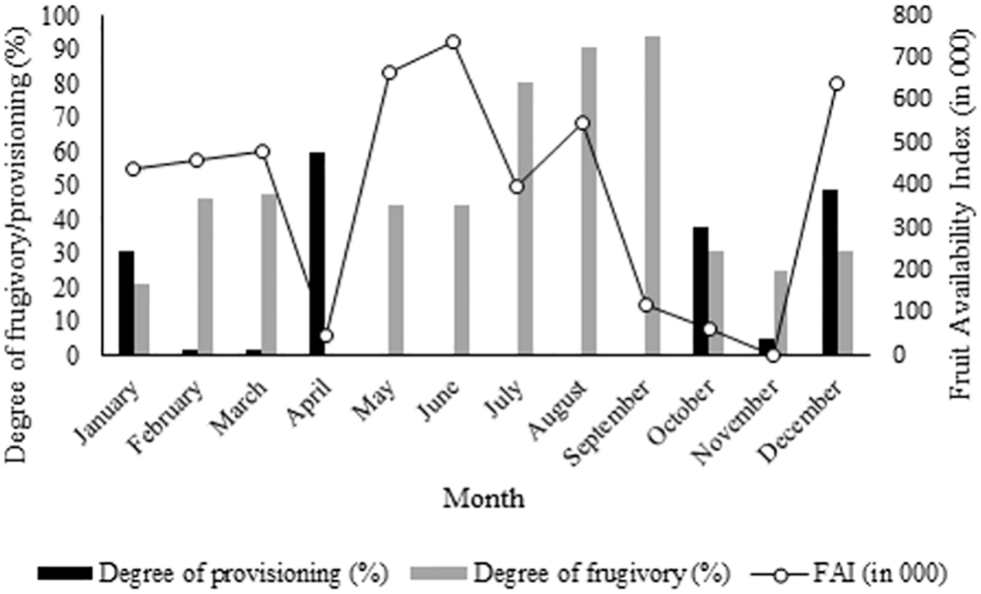
Fruit Availability Index, degree of provisioning and degree of frugivory across the study period.

**Fig 3:**
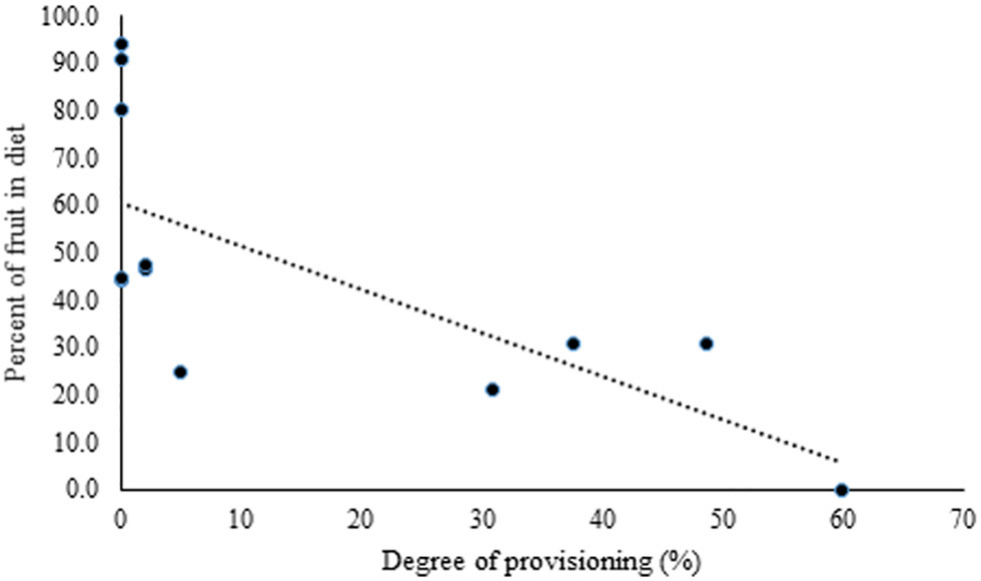
Relationship between degree of provisioning and degree of frugivory (percent of fruit in diet).

The Fruit Availability Index (FAI) ranged from 1688.1 in November to 739,633.4 in June (Mean = 383,993.8 ± SD 249,715.3) (Fig 2). Across the year, the diet of rhesus macaques comprised fruits (46.3%), leaves (30.4%), flowers (3.2%), insects (4.6%) and human subsidised food (15.5%); their dependence on fruits and human subsidised food varied over the months. Provisioning ranged from 2% (February and March) to 60% (April) (Fig 2). The degree of provisioning was not related to FAI (r = -0.3, p = 0.33). Fruits accounted for 70.8% of the diet in the non-provisioning period and 28.8% of the diet during the provisioning period. Frugivory was highest in September (94%), when no provisioning occurred, and zero in April (when provisioning was highest) (Fig 2). Degree of frugivory in the macaques was related to the degree of provisioning (r = -0.71, p< 0.05) (Fig 3) but not to FAI (r = 0.21, p = 0.5).

### Sites of seed deposition

**Fig 4:**
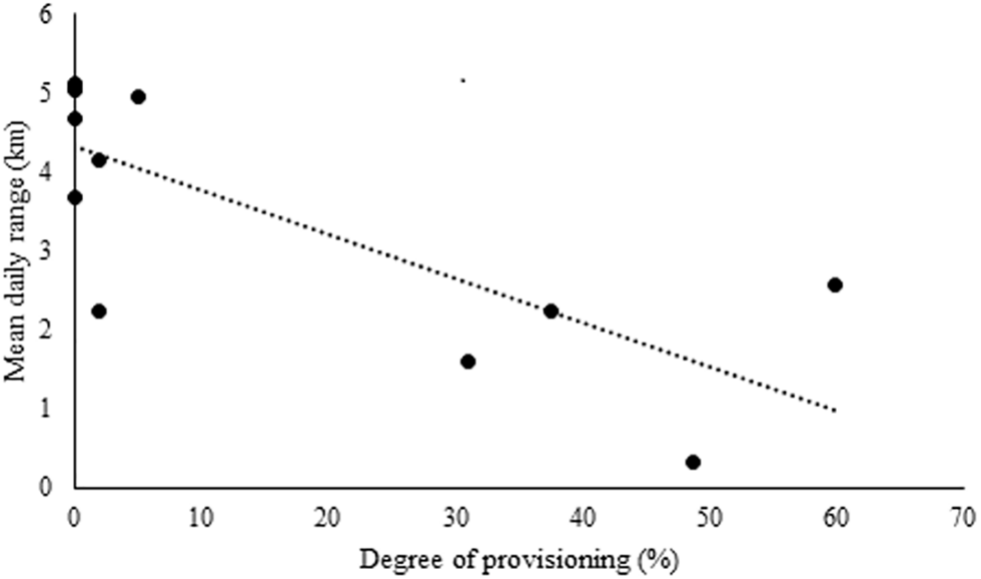
Relationship between degree of provisioning and mean daily range of the study troop.

Across the year, the mean daily range for the study troop was 3.54 km (± SD 1.54, N = 120 days). The mean daily range for each month had a significant negative correlation with the degree of provisioning (r = -0.76, p < 0.01) (Fig 4). The mean daily range was lowest in December (0.32 km) and highest in September (5.12 km). During the non-provisioning period, the mean daily range was 4.72 km (N = 5 months) whereas in the provisioning period it was 2.58 km (N = 7 months).

### Seed handling mechanisms and dispersal events

Overall, rhesus macaques fed on the fruits of 27 species (fruit species henceforth) (Table 1). The number of fruit species fed on in a month ranged from 0 (April) to 14 (August) (Table 2). A significantly greater percentage of seed species were spat out (59.2%) than were swallowed (22.2%) or destroyed (18.5%) (χ^2^ = 30.4, df = 3, p < 0.001) (Table 1).

**Table 1:** Seed fate of species fed on by rhesus macaques.

Species	Family	Spat out	Swallowed	Destroyed
*Acacia auriculiformis*	Fabaceae			x
*Albizia lucida*	Fabaceae			x
*Anogeissus latifolia*	Combretaceae	x		
*Anthocephalus chinensis*	Rubiaceae		x	
*Antidesma diandrum*	Euphorbiaceae	x		
*Artocarpus chaplasha*	Moraceae	x		
*Baccauria sapida*	Euphorbiaceae	x		
*Beilschmedia gammeiana*	Lauraceae	x		
*Careya arborea*	Lecythidaceae	x		
*Chisocheton paniculatus*	Meliaceae	x		
*Elaeocarpus aristatus*	Elaeocarpaceae	x		
*Elaeocarpus varuna*	Elaeocarpaceae	x		
*Ficus benghalensis*	Moraceae		x	
*Ficus benjamina*	Moraceae		x	
*Ficus racemosa*	Moraceae		x	
*Ficus religiosa*	Moraceae		x	
*Gmelina arborea*	Verbeneceae	x		
*Mangifera indica*	Anacardiaceae	x		
*Michelia champaka*	Magnoliaceae			x
*Phyllanthus emblica*	Phyllanthaceae	x		
*Polyalthia simiarum*	Annonaceae	x		
*Populus gamblei*	Salicaceae	x		
*Quercus lancefolia*	Fagaceae			x
*Syzygium formosa*	Myrtaceae	x		
*Talauma hodgsonii*	Magnoliaceae	x		
*Terminalia chebula*	Combretaceae			x
*Zanthoxylum budrunga*	Rutaceae		x	

**Table 2:** Dispersal event ratios for fecal and spat out seeds.

Month	Degree of provisioning (%)	Number of fruit species consumed	No. of fecal samples collected	No. of fecal samples containing seeds	No. of fruit feeding scans	No. of scans in which macaques spat-out seeds	DE_r_		Number of Dispersal Events observed on the road		DS_r_	
							Fecal	Spat out	Fecal	Spat out	Fecal	Spat out
January	30.96	5	49	3	51	5	0.06***	0.1***	2	5	0.67	1***
February	2	6	15	5	111	34	0.33	0.31***	1	2	0.2	0.06
March	2	4	15	5	114	57	0.33	0.5*	1	3	0.2	0.05
April	60	0	6	0	0	0	0*	0***				
May	0	4	23	21	107	71	0.91***	0.67	4	2	0.19	0.03
June	0	10	22	20	107	75	0.91***	0.7*	0	3	0***	0.04
July	0	6	28	20	193	175	0.71***	0.91***	0	13	0***	0.07
August	0	14	17	10	218	218	0.59	1***	3	16	0.3	0.07
September	0	12	10	10	225	149	1***	0.66*	0	11	0	0.07
October	37.62	2	131	39	74	10	0.3***	0.13***	24	7	0.62***	0.74***
November	5	8	29	21	60	0	0.72***	0***	2		0.1	
December	48.71	3	22	0	74	2	0***	0.03***		2		1*

Binomial Test

*p <0.05

** p<0.01

***p<0.001

**Fig 5:**
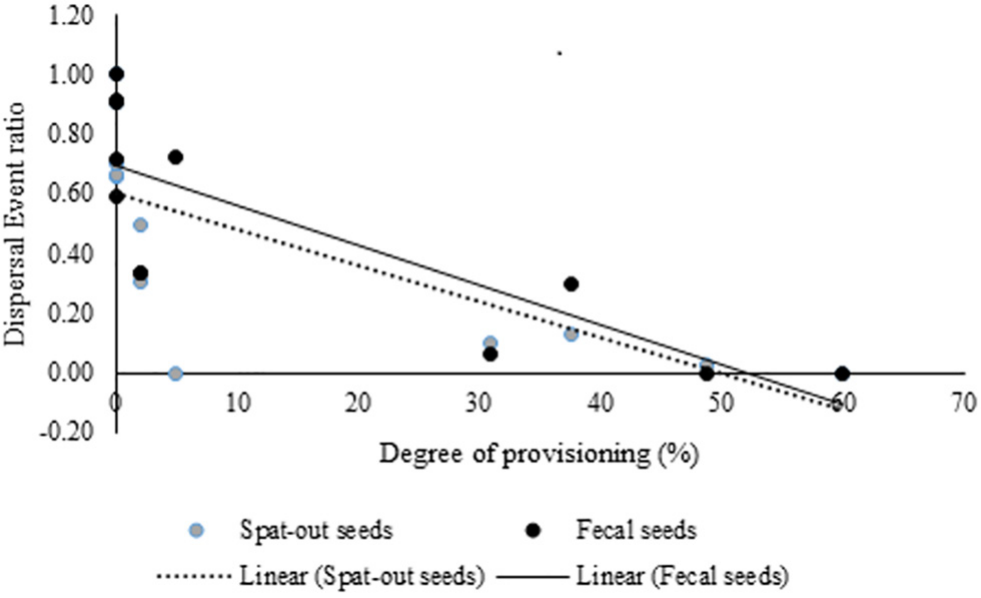
Relationship between degree of provisioning and Dispersal Event Ratio for fecal and spat-out seeds.

Overall, 42% of the fecal samples contained seeds of at least one species. Eighty-one percent (N = 100) of fecal samples in the non-provisioning period contained seeds; only 27.3% (N = 267) of fecal samples in the provisioning period contained seeds (Table 3). Fecal samples from December and April (months with the highest degrees of provisioning) contained no seeds. The DE_r_ for fecal seeds ranged from 0.06 in January to 0.91 in May and June and was negatively correlated with the degree of provisioning (r = -0.81, p < 0.01) (Fig 5) (Table 2).

**Table 3:** Differences in frugivory and seed dispersal activity between non-provisioned and provisioned periods.

Parameters		Non-provisioned period	Provisioned period
Mean daily range (km)		4.72 (N = 5 months)	2.58 (N = 7 months)
Degree of frugivory (%)		70.8 (N = 1200 scans)	28.8 (N = 1680 scans)
Dispersal Events (%)	Fecal seeds	81 (N = 100 fecal samples)	27.3 (N = 267 fecal samples)
	Spat out seeds	81 (N = 850 fruit-feeding scans)	22.2 (N = 484 fruit feeding scans)
Dispersal Events on roads (%)	Fecal seeds	9 (N = 81)	41 (N = 73)
	Spat out seeds	7 (N = 688)	18 (N = 108

We collected a total of 1334 fruit-feeding scans. During the non-provisioning and provisioning periods, 81% (N = 850) and 22.2% (N = 484) of the fruit-feeding scans respectively were associated with seed spitting. (Table 3). Macaques did not spit out any seeds in April (month of highest provisioning), but spat out seeds in all the scans (N = 218) in August (no provisioning). Across the year, the DE_r_ for spat out seeds was 0.6 (N = 1334), and was negatively correlated with the degree of provisioning (r = -0.74, p<0.01) (Fig 5) (Table 2).

**Fig 6:**
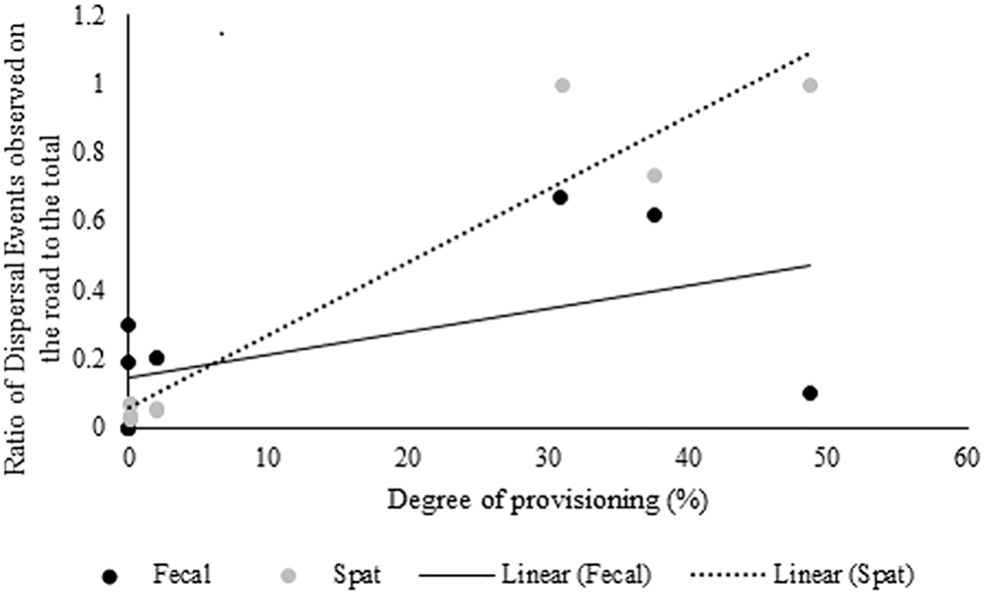
Relationship between ratio of Dispersal Events observed on the road to the total and degree of provisioning for fecal and spat-out seeds.

Fecal seeds were deposited in primary forests (53%), plantations (23%) and on motorable roads (24%) (N = 154). (Table 3). DS_r_ for fecal seeds had a significant positive correlation with the degree of provisioning (r = 0.89, p<0.001) (Fig 6) (Table 2). Forty one percent and 9% of the seed deposition sites were on roads during the provisioning and non-provisioning periods respectively. No fecal seeds were deposited on roads in June, July and September whereas 67% of the deposition sites of fecal seeds were found on roads in January.

Spat out seeds were deposited in primary forests (59%), plantations (33%) and on the roads (8%) (N = 796) (Table 3). During the provisioning and non-provisioning periods, 18% and 7% of the seed deposition sites were on roads respectively. In December and January (provisioning months) all spat-out seeds were deposited on the road. DS_r_ had a significant positive correlation with the degree of provisioning (r = 0.96, p < 0.001) (Fig 6).

## Discussion

Human food subsidies to animals have been implicated in many far-reaching modifications to ecosystem processes and food webs [3]. Yet, the particular impacts of provisioned food entering natural food webs across species, communities or ecosystems remains relatively unknown [2]. Empirical studies dealing with this issue are limited in number [42]. In a natural scenario it is difficult to control the quantum of provisioning or quantify its effects on ecosystem processes; nor is it possible to conduct experimental designs with control conditions and replication trials [20]. Thus it becomes difficult to establish strong links between provisioning and its impact on ecosystem processes. The unusual situation in BTR–the presence of a primate troop that was provisioned for only some months in a year–provided us with a naturally occurring experimental set up, wherein we could test the direct effects of provisioning on an important ecological process, namely seed dispersal.

Seed Dispersal Effectiveness (estimated as the ‘number of new adult plants produced by the dispersal activities of a disperser) [43] is typically measured in terms of (i) number of seeds of different plant species dispersed by the frugivore and (ii) the probability of survival and germination of the dispersed seed and the subsequent production of an adult tree [43]. Our study results highlight the impact of provisioning on the seed dispersal effectiveness of rhesus macaques in two ways. Firstly, a comparison of seed dispersal activities between Troop C (the wild troop [32]) and Troop D (provisioned troop, present study) shows that provisioning affects frugivory levels of the species and seed deposition rates negatively. Troop C diet comprised 79% fruits [32], compared to 46% fruits in Troop D’s diet. For Troop C, all handled seeds were deposited on the forest floor and 50% of monitored seeds germinated during the study period [32]. In contrast, for Troop D, 24% of fecal seeds and 8% of spat out seed deposition sites were on tarmac roads which were unsuitable for germination. Although we did not monitor seeds through to establishment, provisioned macaque troops were clearly less effective as seed dispersers than macaque troops that were completely dependent on natural resources.

Secondly, a comparison of frugivory and dispersal activities between the provisioned and non-provisioned periods of Troop D in the present study also shows that provisioning impacts these behaviours negatively. Provisioning reduced the degree of frugivory in the macaques by 42%, and dispersal activities through defecation and spitting by 54% and 59% respectively. Although the length of our study did not allow us to assess production of adult trees, our results do stress that survival and germination of dispersed seeds were curbed as a consequence of provisioning. The macaques deposited more seeds in areas unconducive for germination during the provisioned period (41% and 18% of the fecal and spat-out seed deposition sites respectively on roads) in contrast to non-provisioned period (9% and 7% for fecal and spat-out seed deposition sites respectively on roads).

Long‐distance dispersal is critical to plant dynamics, being central to population spread, persistence of subpopulation, recolonization and gene flow [43] and dispersal distances for seeds are affected by daily ranges of frugivores [29]. In this study, the mean daily range reduced by nearly 50% during the provisioning period indicating that provisioning may result in shorter dispersal distances. Although we did estimate dispersal distances for individual handled seeds in the present study and conducted germination experiments in the field station, we did not report these results; irrespective of the dispersal distance, deposition on tarmac roads vitiates seed germination. Secondly, although secondary dispersers may rescue some seeds, deposition on roads also results in a higher probability of seed destruction due to moving vehicles, thereby minimising the chances of secondary dispersal.

Primates may resort to feeding on food from anthropogenic sources for one or more reasons: (i) they develop a preference for human food, (ii) natural resource availability is too low to meet the demands of a population, (iii) provisioned food is nutritionally richer and energetically easier to access [12, 44–45]. For example, at the Bandipur National Park in southern India, bonnet macaques *Macaca radiata* usually foraged on natural food sources but recoursed to provisioning in seasons when natural resources were patchily distributed and/or when tourist traffic within the Park was at its peak [44]. In the present study, macaque frugivory was inversely correlated with degree of provisioning, but the degree of frugivory was not related to fruit availability. This could indicate low natural resource availability in the study area; this premise is supported by our data that mean fruit availability in the study area (Damanpur Block) was about 18% lesser than that in the nearby Checko Block area. Accordingly the Troop D in Damanpur Block fed on 27 fruit species, whereas Troop C in Checko Block fed on 49 fruit species.

We could not test if macaques preferred human food subsidies as plant species fed-on preferentially by macaques at other sites (Troop C [32]) (*Antidesma diandrum*, *Artocarpus chaplasha*, *Baccauria sapida*, *Chisocheton paniculatus*, *Elaeocarpus varuna* and *Mangifera indica)* were only available during the non-provisioning period [46]. Further studies on semi-provisioned macaque troops in areas where human food subsidies are accessible throughout the year would throw more light on this matter. Studies involving nutritional analysis of provisioned and natural foods would help establish if macaques feed upon particular kinds of food at different times of the year in order to meet their varying nutritional requirements.

Our studies involving non-provisioned [32] and semi-provisioned rhesus macaques bring to the fore the critical role of macaques as seed dispersers in disturbed ecosystems and how human interventions negatively affect this ecological function. We recommend two initiatives that may help avert macaque dependence on human food subsidies and thereby aid in mitigating conflict and ensure the maintenance of natural ecosystems. One could be the establishment of afforestation programs involving preferred plant species in order to prevent rhesus macaques from gravitating towards human habitations and getting into conflict over shared resources. The second would be a complete cessation of provisioning activities. This however is not an easy task to achieve. During the present study, we observed that although there were several sign-boards in English and the regional languages put up by the State Forest Department along roadsides cautioning tourists against feeding monkeys, many of the tourists did not heed these warnings. Provisioning wild animals is a socio-cultural tradition across much of South and South-East Asia that is deeply entwined with notions of charity and religious piety [7], hence such bans may not serve the purpose. However, educational programmes within BTR and other protected areas of the country informing people about the ill-effects of provisioning would be an important step forward in reducing the practice Such programmes should not only focus on the ecological effects but also on more proximate causes of worry such as animal road-kills, bi-directional disease transmission, heightened conflict etc. They should also encourage residents and tourists to practise better garbage management and to stop feeding wildlife. Finally more stringent vigils by Forest Department officials and imposition of fines on people trying to feed animals may prevent provisioning to a large extent. However, provisioning is not restricted to areas in and around protected forests, but occurs in urban areas as well. Hence we also propose the launch of a nation-wide literacy programme that educates lay citizens on the consequences of feeding wildlife and thereby attempts to bring in an attitudinal change regarding provisioning.

It has been argued that much of the environmental crisis facing us today may be traced to a lack of public understanding of ecosystem processes [47]. Clearly, as the results of our study demonstrate, this is not only applicable to large-scale anthropogenic impacts such as deforestation, habitat disturbance and environmental pollution, but also to seemingly inconsequential and benign actions such as human feeding of wildlife.
